# The Importance of Attending the Columbian Dental Congress

**Published:** 1893-05

**Authors:** 


### Editorial.

### THE IMPORTANCE OF ATTENDING THE COLUMBIAN DENTAL CONGRESS.

To all readers of the dental periodicals, the meeting of the Dental Congress at Chicago, in August, is a subject of interest, as it marks an event which is to demonstrate the advanced state which dentistry has reached in the scientific elements pertaining to it, as well as the growth in its practical development. It may be questioned whether this general interest has excited the profession to a sufficient appreciation of the great value the meeting will have to those who may attend it, and the stimulus it probably will effect upon the succeeding decades. If it be well attended, and if the management shall control the opportunity with its possibilities, it will become a marked event in the history of dentistry.

To this Congress has been invited leading men from all parts of the world. Many of these occupy advanced positions in the countries from which they will come. They inevitably will have views to present in the modes of thought peculiar to them. Papers will be read by these which, from the nature of the occasion, must call out the best efforts of their authors. The men of our own country most nearly identified with scientific efforts will also be there, with whatever they may have prepared to lay before the minds gathered to do honor to the memorable occasion.

The ruling thought of those originating and managing the Congress was, and continues to be, to make it represent the highest professional and best scientific thought of the day. That purpose will continue to govern to the end. This being the leading and the animating motive, it becomes all interested in the welfare of our profession to make arrangements to be present. Neither personal feeling, if any such should exist, nor inconvenience should deter any from being one of the throng to welcome our friends, and to do whatever they may to further advance the position to which the science and art of dentistry has reached.

To those of foreign countries who have been delegated to attend, and to those who may not have had special invitation, the occasion is one which will well repay for the necessary journey, as they will have the largest opportunity to observe the multitudinous devices and methods connected with applied dentistry, which is a field in which, it must be conceded, America is predominant. To those of our own country it is of extreme importance they do not let the event pass without their presence,—not only that they may benefit by the higher features presented, but that the occasion may be inspirited by the influence of numbers united in a common effort. It is also due the managements that a spontaneous response should be given to the labor which the officers of the Congress have been making to insure a successful result.

The plea which some have made, that the Congress is open to depreciation because the invitation to attend has been extended to all qualified dentists, should have no value to deter any from giving the Congress the support of their presence. The intention is to make the meeting an educational one, with a warm international character. It will have much of the characteristics of the World's Fair, of which it is a portion. It is an exposition of the products of mind, and they who have this wealth to display, in so doing honor themselves and ennoble their motives. If only those who are engaged in developing the scientific features of dentistry were to constitute the Congress, it would prove a cold affair, and the proceedings would have little influence over our progress. There must be those to receive instruction as well as those to give it. So let the genius of democracy and the influence of numbers animate the meeting, that its potency may be widely felt.

Let us trust that both home and foreign representatives will be fully present at the Columbian Dental Congress, that the largest benefits may grow out of it.

